# Supplementary material for: The wild life of ticks: Using passive surveillance to determine the distribution and wildlife host range of ticks and the exotic Haemaphysalis longicornis, 2010–2021
Source: Parasit Vectors. 2022 Sep 20;15:331. doi: 10.1186/s13071-022-05425-1 (PMC9487032; doi:10.1186/s13071-022-05425-1)
Supplement: Supplementary file 2 — Additional file 2: Number of host submissions by state. Graphic detailing number of hosts submitted by state. [file 13071_2022_5425_MOESM2_ESM.docx]

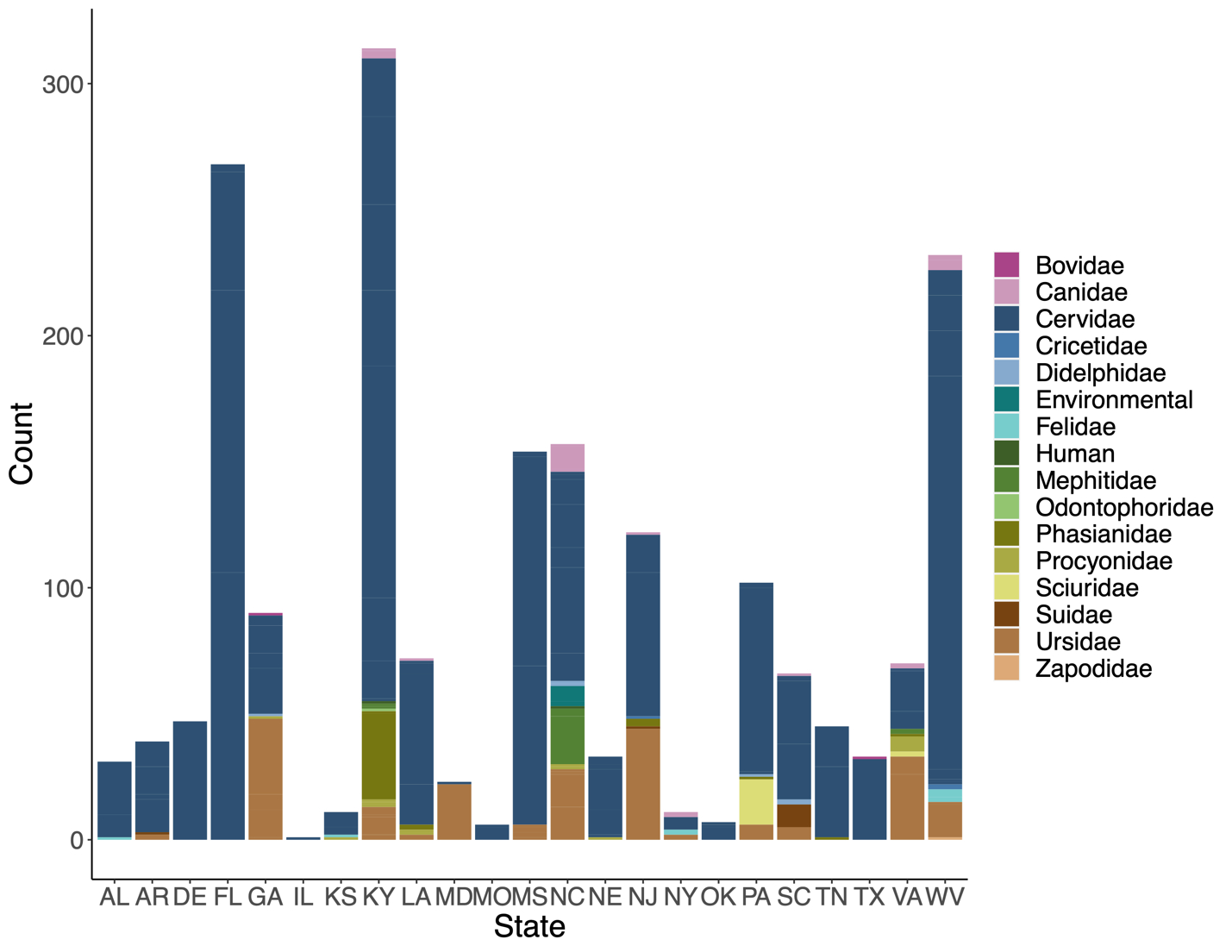


Additional File 2. Host submissions by state, 2010 – 2021. Stacked bar chart represents total number of submissions by state, bars are colored in by number of host group submitted.
